# No radiographic wrist damage after treatment to target in recent-onset juvenile idiopathic arthritis

**DOI:** 10.1186/s12969-019-0362-1

**Published:** 2019-09-04

**Authors:** P. C. E. Hissink Muller, W. G. van Braak, D. Schreurs, C. M. Nusman, S. A. Bergstra, R. Hemke, D. Schonenberg-Meinema, J. M. van den Berg, T. W. Kuijpers, Y. Koopman-Keemink, M. A. J. van Rossum, L. W. A. van Suijlekom-Smit, D. M. C. Brinkman, C. F. Allaart, R. ten Cate, M. Maas

**Affiliations:** 1 Department of Pediatric Rheumatology, Leiden University Medical Center, Leiden, the Netherlands; 2 Department of Pediatrics/Pediatric Rheumatology, Sophia Children’s Hospital Erasmus Medical Center, J6-S, LUMC, PO Box 9600, 2300 RC Leiden, Rotterdam the Netherlands; 3 Department of Radiology and Nuclear Medicine, Amsterdam University Medical Center, Amsterdam Movement Sciences, Amsterdam University Medical Center, Amsterdam, the Netherlands; 4 Department of Pediatric, Immunology, Rheumatology and Infectious Diseases, Emma Children’s Hospital, Amsterdam University Medical Center, Amsterdam, the Netherlands; 5 Department of Pediatrics, Hagaziekenhuis Juliana Children’s Hospital, the Hague, the Netherlands; 6 Department of Pediatric Rheumatology, Amsterdam Rheumatology and Immunology Center location Reade, Amsterdam, the Netherlands; 7 Department of Pediatrics, Emma Children’s Hospital AMC, Amsterdam University Medical Center, Amsterdam, the Netherlands; 8 Department of Rheumatology, Leiden University Medical Center, Leiden, the Netherlands

**Keywords:** Juvenile idiopathic arthritis, Treatment to target, Radiographic outcome, Conventional radiography

## Abstract

**Background:**

To evaluate radiographic progression of patients with new-onset juvenile idiopathic arthritis (JIA) in response to an early, tightly-controlled, treatment-to-target.

**Methods:**

Patients with JIA participating in the BeSt-for-Kids-study, randomized to 3 treatment strategy arms, were eligible if at least 1 conventional wrist-radiograph was available. Bone damage as reflected by carpal length was assessed using the Poznanski-score. The BoneXpert-method was used to determine the Bone Age (BA, > 5 years) and bone mineral density (BMD) of the wrist. These scores were evaluated over time and compared between the treatment arms and mean JADAS10-score using linear mixed models corrected for age and symptom duration.

**Results:**

In 60 patients, 252 radiographs were analysed. Baseline age and symptom duration were different between the arms. No difference in comparison to the healthy reference population was found at baseline for the Poznanski-score (IQR varying from − 0,82; 0.68), nor for BA (varying from − 0.88 to 0.74). Baseline BMD was statistically significantly lower in arm 3 (initial treatment with etanercept and methotrexate) (− 1.48; − 0.68) compared to arm 1 (− 0.84; − 0.04) and arm 2 (− 0.93; 0.15). After treatment to target inactive disease, the Poznanski-scores and the BA remained clinically unchanged, while the BMD in arm 3 improved (*p* < 0.05 vs arm 1).

**Conclusions:**

Recent-onset JIA patients, treated-to-target aimed at inactive disease, showed no signs of radiographic wrist damage (Poznanski-score, BA or BMD) either at baseline or at follow-up, irrespective of treatment arm. A lower BMD at baseline in arm 3, initially treated with methotrexate and etanercept, improved significantly after treatment.

**Trial registration:**

NTR, NL1504 (NTR1574). Registered 01-06-2009.

**Electronic supplementary material:**

The online version of this article (10.1186/s12969-019-0362-1) contains supplementary material, which is available to authorized users.

## Background

Juvenile idiopathic arthritis (JIA) is a potentially chronic disease that comprises 7 categories of childhood arthritis of unknown cause, that persists for more than 6 weeks and starts before the age of 16 [1]. Osteopenia, bony deformity, erosions, and cartilage loss in carpalia, resulting in carpus shortening, can be complications of inflammation in JIA patients [2–5]. Previous studies have shown that early damage on conventional radiography is correlated with functional deterioration and radiographic progression after 5 years [2, 5, 6], and also with smaller chances to achieve clinical remission [7]. Monitoring of radiographic damage progression is therefore important to evaluate treatment effect and predict prognosis. Since joint damage is assumed to be the result of ongoing inflammation, reaching inactive disease as early as possible and thereby preventing structural joint damage and consequently limitations in physical functioning, should be the goal of treatment [8]. This is facilitated by the availability of new effective disease modifying antirheumatic drugs (DMARDs) [9]. In accordance, current JIA treatment recommendations focus on earlier introduction of DMARDs aiming to achieve remission or at least low disease activity [8, 9].

We have recently performed a randomized clinical trial using the treatment-to-target approach in recent-onset JIA patients, comparing 3 strategy-arms with different initial and subsequent treatment steps, aiming at inactive disease, including tapering and stopping DMARD therapy [10, 11]. In this population we studied radiographic wrist damage using the Poznanski-score, at baseline and evaluated whether damage occurred or recovered with the abrogation of inflammation in the 3 strategy-arms. In addition we used the BoneXpert-method to determine the Bone Age (BA) and Bone Mineral Density (BMD) as markers for joint damage [12].

## Methods

### Patient selection

The Best-for-Kids-study (NTR 1574), a multicenter randomized single-blinded clinical trial, was designed to investigate the effectiveness of three different treatment-strategies in newly diagnosed patients with the following JIA categories: oligoarticular JIA, rheumatoid factor (RF) negative polyarticular JIA and juvenile psoriatic arthritis. DMARD-naive patients with a disease duration of less than 18 months were randomized to one of the three treatment arms.

**Fig. 1:**
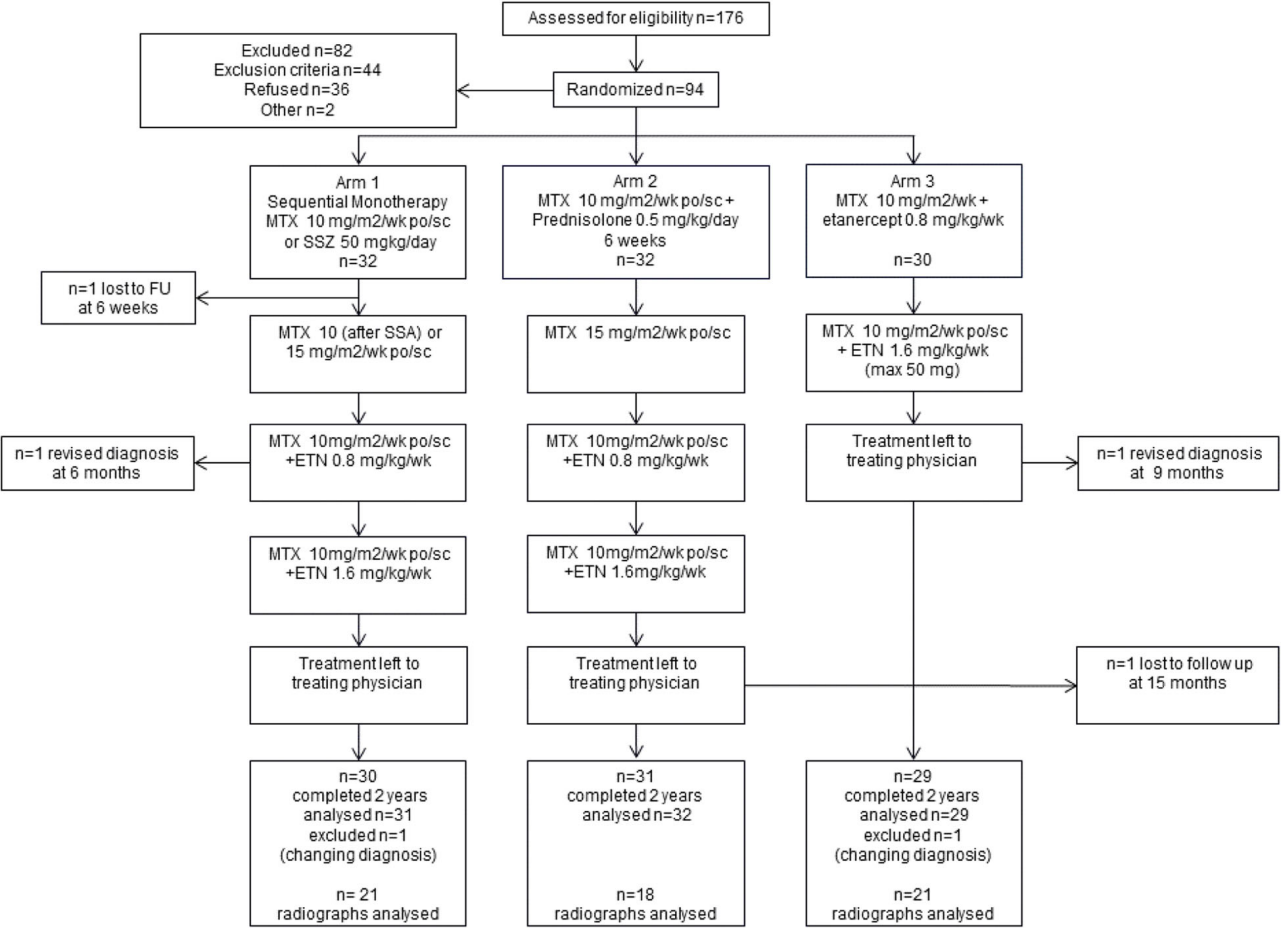
The three treatment strategies compared in the BeSt for Kids study. Flow diagram of the three treatment strategies compared in the BeSt for Kids study; Revised diagnosis were localized scleroderma with arthritis (arm 1) and polyarteritis nodosa (arm 3). See patients and methods section for description of treatment groups. FU = follow-up, SSZ = sulfasalazine, MTX = methotrexate, ETN = etanercept, po = orally, sc = subcutaneous

Patients in arm 1 were treated with initial monotherapy with methotrexate (MTX) or sulfasalazine (SSZ); patients in arm 2 were treated with initial MTX and prednisone bridging and patients in arm 3 were initially treated with etanercept and MTX. Patients were treated to target, aimed at inactive disease, with three-monthly assessments. If predefined targets of suppression of inflammation were not met, treatment was intensified, as can be seen in Fig. 1, with subsequent treatment-steps, including etanercept also in arm 1 and 2. In case of at least 6 months of inactive disease, treatment was tapered. The current sub-analysis was done in all patients who had radiographs of one or both hands obtained at study inclusion (with a range of maximum 4 months before) or at any follow-up visit up to 40 months. Radiographs of hands and wrists were encouraged at baseline, year 1 and year 2. In practice, physicians were reluctant to do this if there was no local arthritis. Juvenile Arthritis Disease Activity Score (JADAS)10-scores were available from all the patients [11]. To investigate the effect of the relatively fast changing disease activity on slower changing radiological outcome parameters, we have used mean JADAS10-scores over 2 years’ time as a predictor for the radiological outcomes.

The BeSt-for-Kids-study was approved by the Institutional Review Board at Leiden University Medical Center and written informed consent was obtained from all participants before enrollment.

### Radiographic scoring

**Fig. 2:**
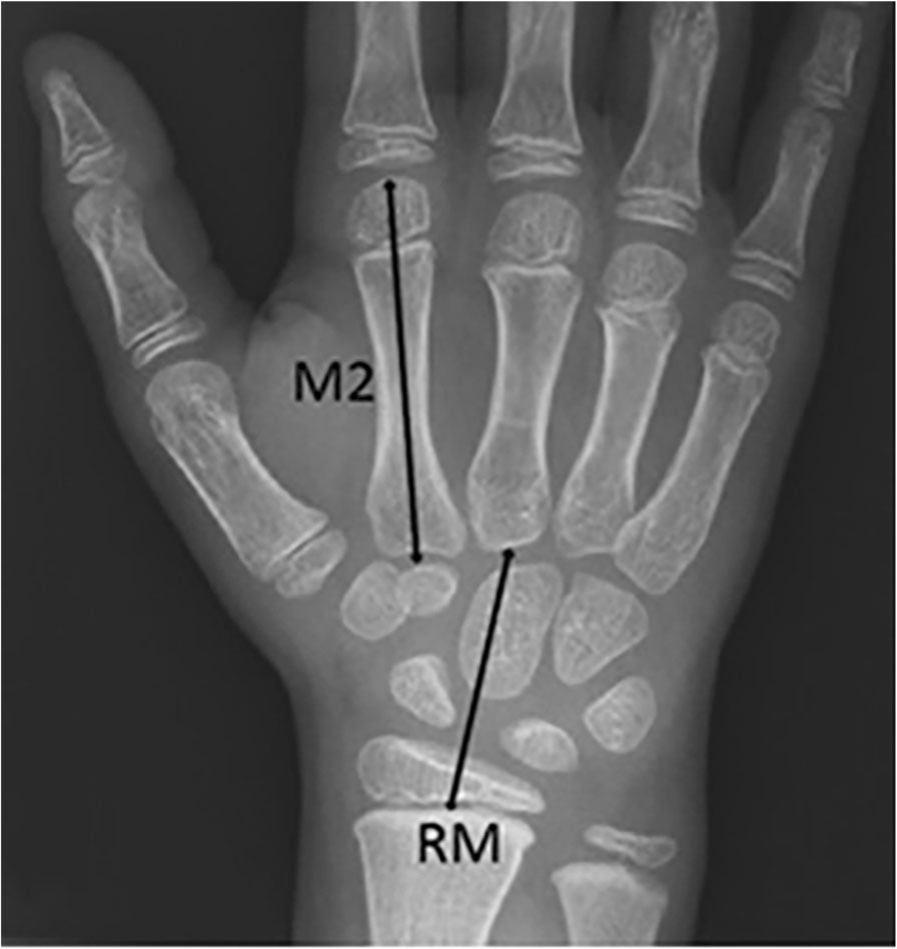
Poznanski measurements used to determine the RM/M2 score. RM = radiometacarpal length; M2 = length of the second metacarpal

All radiographs were anonymized and randomized by an independent computer-technician, and then evaluated using two different scoring methods: the Poznanski-score [13] and the BoneXpert-method. When radiographs of both wrists were available, scores of both wrists were included. The Poznanski-score was used to measure carpal size, and was calculated as the mean score of 2 independent readers (DS and WB), who were unaware of clinical data. Open growth plates are necessary to determine the Poznanski-score. The radiometacarpal length (RM, defined as the line from the mid-growth plate of the radius to the center of the proximal end of the third metacarpal) and the length of the second metacarpal (M2, defined as the maximum length of the second metacarpal as defined by Garn [14]) were measured, in millimeters using RadiAnt DICOM viewer version 2.2.8, as shown in Fig. 2. Poznanski’s gender-specific formulas were used to calculate the expected RM for the observed M2 [13]. The difference between expected and measured RM was then calculated and converted into a Z-score [13], which represents the number of standard deviations that the observed RM diverges from the expected RM. A negative Z-score indicates delayed growth in the radiometacarpal bones with loss of cartilage or loss of joint space as potential causes, whereas a positive Poznanski-score may indicate growth acceleration, a phenomenon thought to be caused by early ossification of carpal bones under influence of chronic hyperemia and inflammation [15]. Radiographic bone damage progression was determined by calculating the change in Z-score between the baseline and follow-up radiographs.

Next, all radiographs were imported as DICOM-files in the BoneXpert-software for automatic assessment of the BA (using the average Greulich and Pyle bone age [16]), and BMD (BoneXpert Version 2.1.0.12; Visiana, Holte, Denmark). The software generates Z-scores of the BA computed relative to provided scores of healthy children of equal gender, age (> 5 years) and ethnicity. A negative Z-score for BA reflects a delayed bone maturation [12, 17] whereas a positive Z-score reflects enhanced focal maturation, also possible due to inflammation [15]. The BMD is automatically determined by measuring the amount of cortical bone in the shafts of metacarpal 2–4. The Z-score of BMD is computed compared to provided scores in healthy children of the same bone age and gender. A negative Z-score indicates a diminished BMD. For the Z-scores, the normal population has a normal (Gaussian) distribution around 0.

### Statistical analyses

The single measure intra-class correlation coefficient (ICC) and Bland-Altman plots with 95% Confidence Interval (CI) were used to determine the agreement of measurements of Poznanski between the two observers and to determine the inter-reader reliability. Baseline characteristics were compared using one-way analysis of variance, Kruskal-Wallis tests or Pearson Chi-square tests, as appropriate. Linear mixed model (LMM) analyses were performed to evaluate the Z-scores of the Poznanski-score, Bone Age and BMD over time between the 3 treatment groups. LMM was also used to evaluate the different Z-scores over time for the mean JADAS-10 score over 2 years’ time, since we assumed that average disease activity over 2 years’ time could have an effect on slower changing variables like Poznanski score, Bone Age and BMD. We assumed a multilevel structure of measurements over time (level 1), nested within hands (left or right, level 2), nested within patients (level 3), and added a random intercept and slope to take into account correlations of measurements performed within the same hand within the same patient and differences in time periods between the different radiographs. For the Poznanski-score, the model was adjusted for the potential baseline confounders age and duration of symptoms. Since the BA and BMD account for age in itself, the models for BA and BMD were adjusted for duration of symptoms only. Multiple imputation using package mice in software package R was used to deal with missing values for symptom duration and JADAS10-score, with *n* = 10 imputed data sets [11].

For all statistical analyses a *p*-value< 0.05 was considered statistically significant. A deviation of > 1 in Z-score, indicating a deviation >1SD from the mean in a normal population, was arbitrarily defined as clinically relevant [17]. Statistical analyses were performed with SPSS version 23 software (SPSS, Chicago, IL., USA) and Stata SE version 14 (StataCorp LP).

## Results

### Patients

Baseline characteristics of the included patients are presented in Table 1. Patients in arm 3 were younger and had longer symptom duration than patients in arm 1 and 2. Nine patients with radiographs initially did not have wrist arthritis, six patients never had wrist arthritis clinically.

**Table 1:** Baseline characteristics of the patients selected from the original 3 arms

	Arm 1 Sequential monotherapy (*n* = 21/31)	Arm 2 Combo MTX + 6wks Prednisone (*n* = 18/32)	Arm 3 Combo MTX + etanercept (*n* = 21/29)	*P*
Age (years), median (IQR)	8.2 (4.1;10.2)	7.9 (5.7;11.7)	6.2 (3.8;10.4)	< 0.001
Symptom duration (months), median (IQR)	7.8 (4.2;11.3)	5.3 (2.6;6.1)	8.5 (4.2;12.1)	0.015
ANA pos, n (%)	8 (38)	6 (33)	8 (28)	0.94
Female, n (%)	14 (66.7)	9 (50)	15 (71.4)	0.36
JIA Category:				0.90
Oligo, n (%)	3 (14.3)	2 (11.1)	2 (9.5)	
Poly, n (%)	17 (81)	14 (77.8)	18 (85.7)	
Psoriatic, n (%)	1 (4.8)	1 (5.6)	1 (4.8)	
VAS physician, mean ± SD (mm)	43.6 ± 15.7	54.0 ± 17.0	52.9 ± 17.5	0.44
VAS patient/parent, mean ± SD (mm)	53 ± 17.1	56.8 ± 23.4	55.2 ± 24.9	0.31
CHAQ, mean ± SD	0.95 ± 0.7	1.1 ± 0.6	1.0 ± 0.6	0.88
No. active joints, median (IQR)	6 (4.5;14.5)	8 (5.8;11.5)	8 (5.5;11.5)	0.56
No. limited joints, median (IQR)	2 (0.5;4)	1.5 (0.8;3.3)	3 (2.5;5.5)	0.68
ESR, median (IQR) (mm)	6 (2;12)	6 (3.5;32)	9 (6;31.5)	0.28
JADAS-10 mean ± SD (0–40)	16.7 ± 4.5	19.6 ± 5.1	19.1 ± 5.8	0.24
Z-score Poznanski median (IQR)^1^	−0.45 (− 0.70;0.56)	−0.19 (− 0.57;0.68)	−0.61 (− 0.82;0.17)	0.056
Z-score Bone Age mean (CI)^2^	−0.38 (− 0.88;0.11)	0.51 (0.28;0.74)	−0.43 (− 0.82;−0.04)	0.001
Z-score BMD mean (CI)^3^	−0.44 (− 0.84;−0.04)	−0.39 (− 0.93;0.15)	−1.08 (− 1.48;−0.68)	0.03
Wrist arthritis, inclusion (%)	19/21 (90)	14/18 (78)	18/21 (86)	
Wrist arthritis, follow-up (%)	17/21 (81)	14/18 (78)	18/21 (86)	
Wrist arthritis, inclusion or follow-up (%)	21/21 (100)	14/18 (78)	19/21 (90)	

*JIA* juvenile idiopathic arthritis, *oligo* oligoarticular JIA, *poly* polyarticular JIA, *IQR* interquartile range, *VAS* Visual Analogue Scale, *ANA* antinuclear antibody, *RF* rheumatoid factor, *SD* standard deviation, *CI* confidence interval, *CHAQ* Child Health Assessment Questionnaire, *No* number, *ESR* Erythrocyte Sedimentation Rate, *JADAS* Juvenile Arthritis Disease Activity Score, *BMD* Bone Mineral Density, *BA* Bone Age (both using BoneXpert method) Z-scores were based on all available radiographs, including left and right hand radiographs. 1: *n* = 35 in arm 1, *n* = 25 for arm 2, *n* = 31 for arm 3; 2: *n* = 16 for arm 1, n = 18 for arm 2 and *n* = 18 for arm 3, 3: *n* = 33, *n* = 25 for arm 2 and *n* = 32 for arm 3. n = amount of X-rays

Of the original 94 patients included in the BeSt-for-Kids cohort, 75 patients had at least 1 hand radiograph available. Overall, 268 radiographs were available. Sixteen radiographs, made outside the selected time frame, were left out, leaving 252 radiographs (*n* = 127 of the left hand and *n* = 125 of the right hand). Of these 92 (in 47 patients) were taken at baseline (with a window of 4 months before and 3 month after inclusion) and 160 (in 52 patients, 27 patients had more than 2 radiographs) during follow-up. Fourteen patients had closed growth plates at baseline and were left out, one patient left the study due to changing diagnosis and was not included in the current analysis [18]. A flow chart of the patient selection process is provided (Additional file 1).

Sixty patients with 252 radiographs (85 in arm 1, 79 in arm 2 and 88 in arm 3) were eligible for scoring by the Poznanski-method and BMD. For analysis of the BA 196 radiographs of 49 patients (65 in arm 1, 67 In arm 2, 64 in arm 3) were eligible, while 56 X-rays (20 in arm 1, 12 in arm 2 and 24 in arm 3) of 11 patients could not be scored because patients’ age was < 5 years.

#### The Poznanski-score

For the Poznanski-score the inter-observer correlations were 0.996 for RM and 0.999 for M2. The intra-observer correlations were ≥ 0.996 for all measurements. Additional file 2 provides the Bland-Altman-plots.

**Fig. 3:**
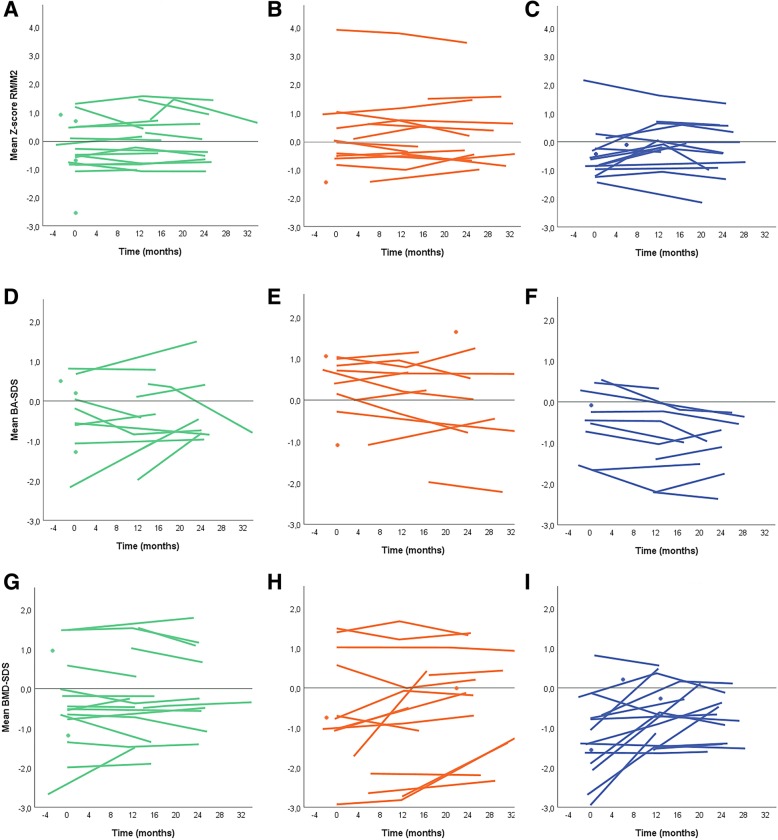
**a**,**b**,**c** – Poznanski-score depicted in Z-scores of RM/M2 ratio. **a** represents patients in arm 1, **b** represents patients in arm 2, **c** represents patients in arm 3. **d**,**e**, **f**- Bone Age depicted in Z-score. **d** represents patients in arm 1, **e** represents patients in arm 2, **f** represents patients in arm 3. **g**,**h**,**i**. Bone Mineral Density depicted in Z-scores. **g** represents patients in arm 1, **h** represents patients in arm 2, **i** represents patients in arm 3. Each graph line represents one individual patient from baseline to follow-up. Each dot represents one patient with a single radiograph available

**Fig. 4:**
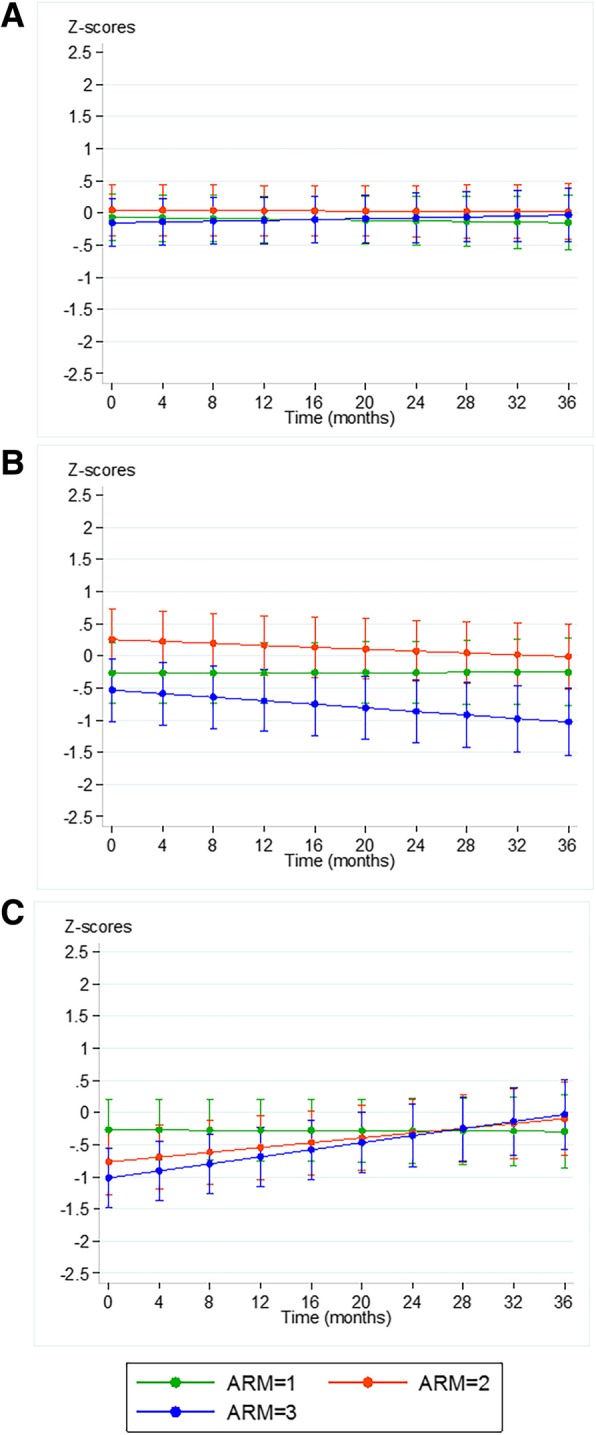
**a**: Predicted Z-score RM/M2 over time, **b**: Predicted Z-score Bone Age over time, **c**: Predicted Z-score BMD over time. All predictions are from Linear Mixed Models, corrected for age and symptom duration for Poznanski score, corrected for symptom duration for BA and BMD. BA = Bone Age, BMD = Bone Mineral Density

At baseline, Poznanski-scores were comparable to those in healthy children, with a median Poznanski-score of − 0.45 (− 0.74–0.45). Over time, overall no significant change in Poznanski-score, unadjusted nor after adjusting for age and symptom duration, was observed, and there were no differences between the 3 arms (see Figs. 3 and 4 for observed and predicted changes in Poznanski-score, BA and BMD Z-scores per arm). The outlier in Fig. 3b with a high Poznanski-score (Z-score 3,9) is a competing mountain-biker.

#### Bone age

At baseline, the mean BA Z-score was 0.04 (-0.58; 0.67) for the entire group, similar to the normal reference population. Baseline scores in arm 3 were significantly lower than in arms 1 and 2, but still within the normal range (1 SD from 0). Over time there was a decrease in BA in arm 3 (arm 3 versus arm 1 *p* = 0.024, β = 0.014 (95%CI -0.002; 0.027) which remained within the normal range (Fig. 4).

#### Bone mineral density

At baseline, the mean BMD Z-score was − 0.65 (− 0.90; − 0.40) for the entire group, with statistically significantly lower baseline BMD in arm 3 compared to the normal reference population. Over time the BMD, adjusted or unadjusted for symptom duration, remained unchanged in arm 1, showed a trend for increase in arm 2 and significantly increased in arm 3 (*p* < 0.001 for arm 3 versus arm 1, β = − 0.028 (95% CI -0.043; − 0.013).

Tables with detailed results of the LMM of the Poznanski-score, BA and BMD are presented in Additional file 3. Results comparing all left with all right hands, both at baseline and during follow-up, were not statistically different for Poznanski-score (*p* = 0.809, BA (*p* = 0.825) nor BMD (*p* = 0.404). Six patients with radiographs never had clinical inflammation of wrists. Sensitivity analyses excluding these patients showed similar results to the main analysis (Additional file 4A). Since we included *n* = 7 patients with oligoarthritis and *n* = 3 patients with psoriatic arthritis, numbers are too small to analyse these groups separately. Sensitivity analysis of the polyarticular subgroup only, showed similar results (Additional file 4B).

#### Effect meanJADAS10-score over time on Poznanski, Bone Age and BMD

To investigate whether mean JADAS10-score over time correlated with any of the radiological outcomes, we have performed separate analyses for the 3 radiological outcome measures. Mean JADAS10-score over time did not influence Poznanski score, [β (95% CI) 0.0010 (− 0.00038; 0.0024), *p* = 0.154], Bone Age [β (95% CI) -0.00017 (− 0.0018; 0.0014), *p* = 0.84] or BMD [β (95% CI) 0.00069 (− 0.0012; 0.0026), *p* = 0.48]. Tables and graphs from this analysis are reported in Additional file 4C.

## Discussion

Our study is the first to describe longitudinal radiological outcomes of a tightly controlled treat-to-target approach, aimed at inactive disease during 24 months of treatment, in recent onset poly- and oligoarticular JIA patients. Despite a symptom duration of mean (SD) 7.6 (4.9) months and a JADAS-10 of 18.7 (5.6), at baseline, we found no significant differences in Poznanski-score and BA (as measured by the BoneXpert-method) of wrist radiographs compared to healthy children. Only in arm 3 BMD as measured by the BoneXpert-method was significantly lower than the normal reference population. After 24 months of treatment, there was no deterioration in any of the scores and in arm 3 BMD had statistically significantly improved. Mean JADAS10-scores over time were not associated with any of the radiological outcomes in this analysis.

Combined with rapid suppression of symptoms of active arthritis, prevention of damage is an important treatment goal in JIA. Damage has been most notably found in patients with longstanding and/or seropositive polyarticular JIA, but may also occur in seronegative polyarticular JIA and oligoarticular JIA [19–21]. As has been shown in rheumatoid arthritis, it is thought likely that, also in JIA, damage progression is driven by inflammatory processes. Assessing damage in patients who are in very different phases of joint development can be challenging. In growing children, cartilage thinning, delayed or accelerated growth and reduced bone mineral density rather than bony erosions and joint space narrowing may indicate damage. Decreased bone age often reflects delayed bony maturation in JIA [22, 23] but also increased focal bone maturation can be a result of joint inflammation [15].

Compared to older cohorts [12, 24] or recent cohorts with longer disease duration [17], we found little damage at baseline in this cohort with recent-onset disease. Since we did not include patients with RF-positive polyarticular JIA, this could be a mildly affected cohort although initial JADAS10 scores were similar to other cohorts [25]. In addition we found no significant damage progression. This is possibly due to our strategy of tightly controlled treatment-to-target aiming at inactive disease in all 3 treatment arms, resulting in rapid suppression of inflammation in most patients, without significant differences between the strategy-arms after 24 months. Only in arm 3 there was an initial greater clinical improvement [10]. We cannot rule out an additional positive effect of use of etanercept, in all patients in arm 3, and in many in arms 1 and 2 after they failed to achieve remission on initial treatment with methotrexate (with or without temporary prednisone). Previous studies suggest that treatment with methotrexate cannot prevent joint damage progression whereas use of biologic DMARDs (used as initial treatment in our arm 3) may be more successful, although data are limited [26, 27]. Apart from strategy, we did not find an effect of mean JADAS10-score over time, possibly in all patients due to rapid suppression of inflammation, therefore inhibiting the disease to have time to create damage.

To score differences in potentially little damage, we needed a sensitive scoring method. Conventional radiography has proven to be a useful modality to monitor wrist damage of JIA patients [2, 4, 5, 13, 28–31]. Several methods, like the Dijkstra-score [31], modified Sharp van der Heijde-score [5, 19] the modified Larsen-score [4, 32] and the Steinbrocker-scale [33, 34] have been developed to evaluate radiographic damage to the osteochondral structures of the wrist and hand. The Dijkstra composite-score is limited in the grading of changes for severity over time [35]. We stopped using the modified Sharp van der Heijde for pediatric assessment of joint damage [5] as it proved too difficult to uniformly score subtle changes in joint space narrowing, bony erosions and bone deformity, as was recognized previously [35]. Magnetic resonance imaging (MRI) and ultrasound (US) are suitable for monitoring disease activity for evaluating treatment response, and may also detect damage [36]. However, interpretation of MRI findings of the osteochondral domain in JIA patients is challenging due to characteristics of the growing skeleton, in particular in hand and wrist joints. Bone marrow edema and bony depressions are also frequently seen on MRI in wrists of healthy children [37–40]. Until now, no optimal method has been found to differentiate pathological and standardized age-specific findings in healthy children on MRI and US which limits their use to accurately assess damage and damage progression in the wrist of JIA patients.

The Poznanski-score, which measures relative carpal length on radiographs of the wrist, is able to detect deviating growth in absence of distinct joint space narrowing or erosions [13]. A disadvantage of the Poznanski-score is that it requires open growth plates, which caused ineligibility in 14 of our patients, and unreliability in case of carpometacarpal erosions which hampers discriminating bony ends, which did not occur in our cohort. In addition, we used the relatively new BoneXpert method to score Bone Age and BMD, which, compared to a healthy reference population, can indicate damage due to inflammation.

The BoneXpert method, based on digital X-ray radiogrammetry (DXR), allows to determine the Bone Age and BMD compared to a normal reference population, at lower costs and with lower radiation than manually comparing the hand radiograph with images in the atlas by Greulich and Pyle [16] and than measuring BMD by Dual Energy X-ray Absorptiometry (DXA) [41]. The BMD measurement by BoneXpert is corrected for the size of the cortical bones to compensate for the high variation in stature of growing children, in contrast to DXA. Previous studies have reported on delayed bone maturation as reflected by negative Z-scores for Bone Age [12, 17]. These studies had included patients with more severe or longstanding active disease. However, Borzutzky and others have warned previously, that determining bone age can be challenging in JIA due to accelerated maturation [15, 42].

In JIA patients, BMD is often reduced [12, 17, 43–45]. BMD was significantly lower at baseline in arm 3 (− 1.1 SD, (− 1.48; − 0.68)). This could indicate longstanding or more severe disease. Indeed symptom duration in arm 3 was slightly longer than in arms 1 and 2, although JADAS-10 scores at baseline were similar in the 3 arms. Possibly as a result of rapid and sustained suppression of inflammation, BMD improved significantly over time in arm 3. A previous study also reported improvement of BMD after therapy [45]. It is speculated that this improvement is due to the anti-inflammatory effect of DMARD treatment [46], more specifically due to etanercept [47, 48]. However, no comparison cohort is available to prove that the treatment-to-target approach is responsible for a better radiological outcome.

Future studies are needed to delineate the effect of the treatment-to-target concept on improving bone health as reflected by bone maturation and BMD in JIA.

Our study has some limitations. Although comparable with other studies [2, 26, 49] in children with JIA, we had a relatively small sample size (*n* = 60), and we may have lacked power to detect small differences. In previous studies, results were based on clinically inflamed wrists only. Since we have examined also 6 patients with wrist radiographs of unaffected wrists, in this study we may have underestimated damage, although sensitivity analyses excluding patients who never had any clinical wrist arthritis over 24 months showed similar results. It remains to be determined whether joint damage is mainly due to local inflammation or (also) to systemic inflammatory processes of JIA.

Due to our choice of scoring methods, patients were excluded who had closed growth plates. Also we disregarded results of radiographs made outside the selected time frame. Follow-up time was relatively short compared to previous cohorts. However, often radiographic damage is expected to occur within the first one or two years [2]. Finally, determination of bone health by BoneXpert software needs further validation, including further comparison with existing methods for the determination of BMD in JIA patients [41, 50, 51].

## Conclusions

We conclude that in our cohort of patients with recent-onset JIA who were treated-to-target aiming at inactive disease, wrist-radiographs showed neither damage according to Poznanski at baseline, nor progression after 2 years. Bone age was within normal values at baseline and after follow-up. In arm 3, BMD was lower at baseline but improved significantly towards normalization during treatment. We propose that with earlier start of treatment and treatment to target, the focus of current treatment regimens shifts to damage prevention rather than suppression of damage progression. This will likely also prevent long-term disability. Future JIA-cohorts with more patients and longer follow-up are warranted to confirm these promising results for children with JIA.

## Additional files

Additional file 1: Flow chart of patient selection process for the Poznanski-score. (DOCX 23 kb)

Additional file 2: Bland-Altman plots with 95% limits of agreement. (DOCX 245 kb)

Additional file 3: LMM for Poznanski, BA and BMD adjusted for age and/or symptom duration. (DOCX 14 kb)

Additional file 4: A Sensitivity analysis of patients with wrist arthritis, without *n* = 6 with never wrist arthritis. B Sensitivity analysis of patients with polyarticular JIA. C LMM for mean JADAS10 score in relation to Poznanski, BA and BMD. (DOCX 804 kb)

## Data Availability

The datasets used and/or analyzed during the current study are available from the corresponding author on reasonable request.

## Abbreviations

BMD: Bone Mineral Density
CI: Confidence Interval
CR: Conventional Radiography
DMARD: Disease Modifying Antirheumatic Drug(s)
ICC: Intra-class correlation coefficient
JIA: Juvenile Idiopathic Arthritis
M2: Second metacarpal
MRI: Magnetic resonance imaging
MTX: Methotrexate
RF: Rheumatoid Factor
RM: Radiometacarpal length
RMexp: Expected radiometacarpal length
RMobs: Observed radiometacarpal length
SSZ: Sulphasalazine
US: Ultrasound
