# Supplementary material for: No radiographic wrist damage after treatment to target in recent-onset juvenile idiopathic arthritis
Source: Pediatr Rheumatol Online J. 2019 Sep 4;17:62. doi: 10.1186/s12969-019-0362-1 (PMC6727344; doi:10.1186/s12969-019-0362-1)
Supplement: Supplementary file 1 — Flow chart of patient selection process for the Poznanski-score. (DOCX 23 kb) [file 12969_2019_362_MOESM1_ESM.docx]

Additional file 1 Flow chart of patient selection process for the Poznanski-score

|  | Inclusion | Exclusion |
| --- | --- | --- |
| Identification | BeSt for Kids cohort  (n=94)  Patients with hand radiographs  (n=75) | No hand radiographs (n=19) |
| Scoring | Patients eligible for scoring  (n=61) | Closed growth plates at baseline radiograph (n=14) |
| Analysis | Patients eligible for analysis  (n=60)   - At least 2 radiographs available (n=39) - (Closed growth-plate on follow-up (n=3)*) - Baseline radiograph available only (n=8)* - Follow-up radiograph(s) available only (n=10) | Patients excluded  (n=1)   - Changing diagnosis (n=1) |

*One radiograph moment used for analysis
