# Supplementary material for: No radiographic wrist damage after treatment to target in recent-onset juvenile idiopathic arthritis
Source: Pediatr Rheumatol Online J. 2019 Sep 4;17:62. doi: 10.1186/s12969-019-0362-1 (PMC6727344; doi:10.1186/s12969-019-0362-1)

Additional file 2 Bland-Altman plots with 95% limits of agreement


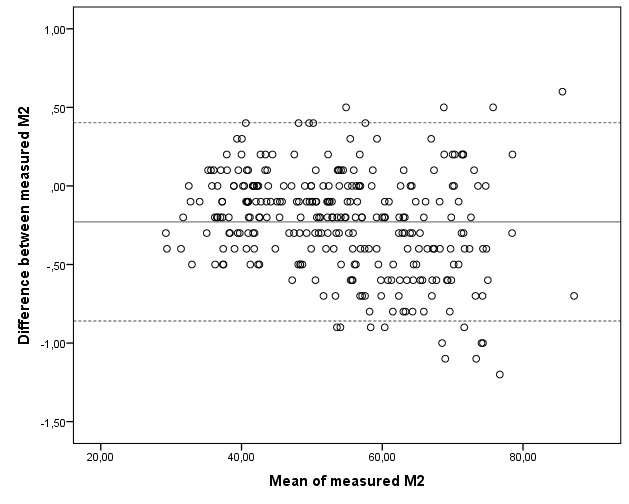

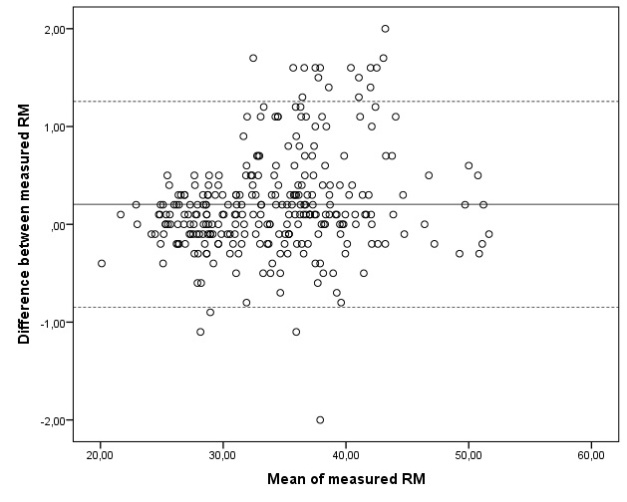
Interreader reliability RM and M2


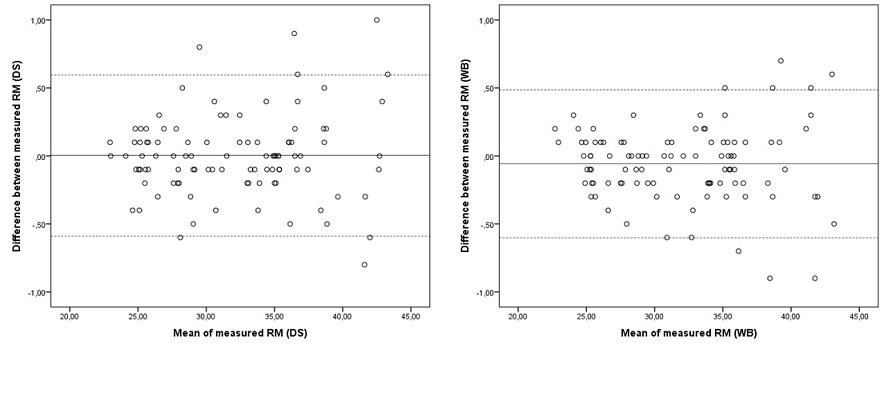
Intra-reader reliability RM and Intra-reader reliability M2


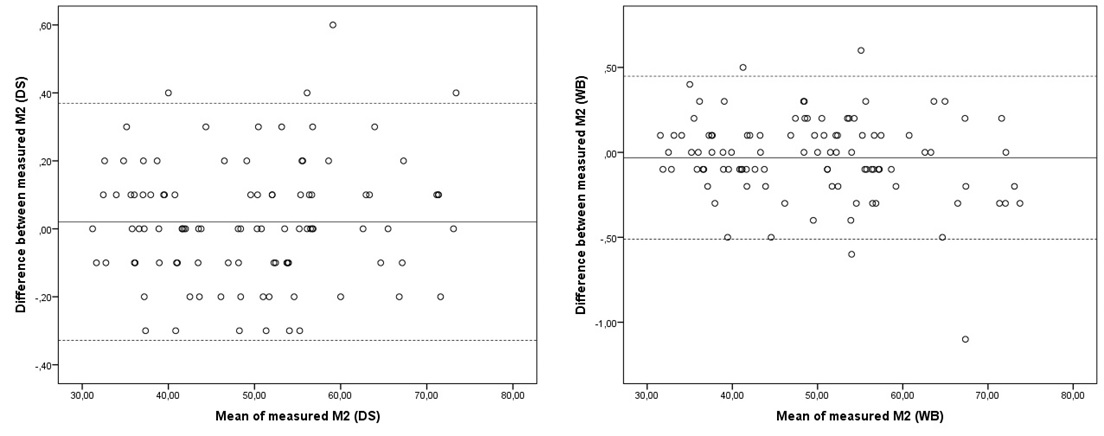

Supplement: Supplementary file 2 — Bland-Altman plots with 95% limits of agreement. (DOCX 245 kb) [file 12969_2019_362_MOESM2_ESM.docx]
