# Supplementary material for: No radiographic wrist damage after treatment to target in recent-onset juvenile idiopathic arthritis
Source: Pediatr Rheumatol Online J. 2019 Sep 4;17:62. doi: 10.1186/s12969-019-0362-1 (PMC6727344; doi:10.1186/s12969-019-0362-1)
Supplement: Supplementary file 3 — LMM for Poznanski, BA and BMD adjusted for age and/or symptom duration. (DOCX 14 kb) [file 12969_2019_362_MOESM3_ESM.docx]

**Additional file 3**

**Table 2, LMM for Poznanski, BA and BMD adjusted for age and/or symptom duration**

| **Patients** | **60** |  |  |  |  |
| --- | --- | --- | --- | --- | --- |
| **X rays** | **117** |  |  |  |  |
| **A Poznanski adjusted for age and symptom duration** | | | | | |
|  | | **Adjusted** |  | **Unadjusted** |  |
|  | | **Β (95% CI)** | **P-value** | **Β (95% CI)** | **P-value** |
| Arm 3 | | ref | - | ref | - |
| Arm 2 | | 0.20 (-0.35; 0.75) | 0.47 | 0.36 (-0.26; 0.98) | 0.26 |
| Arm 1 | | 0.09 (-0.43; 0.61) | 0.74 | 0.21 (-0.39; 0.81) | 0.49 |
| Time | | 0.0033 (-0.004; 0.011) | 0.42 | 0.0035 (-0.004; 0.012) | 0.39 |
| Arm 3 * Time | | ref | - | ref | - |
| Arm 2 * Time | | -0.005 (-0.015 ; 0.007) | 0.47 | -0.005 (-0.016; 0.007) | 0.42 |
| Arm 1 * Time | | -0.006 (-0.017; 0.006) | 0.34 | -0.006 (-0.017; 0.006) | 0.32 |

**Bone Age**

| **Patients** | **41** |  |  |  |  |
| --- | --- | --- | --- | --- | --- |
| **X rays** | **80** |  |  |  |  |
| **B Bone Age adjusted for symptom duration** | | | | | |
|  | | **Adjusted** |  | **Unadjusted** |  |
|  | | **Β (95% CI)** | **P-value** | **Β (95% CI)** | **P-value** |
| Arm 3 | | ref | - | ref | - |
| Arm 2 | | 0.78 (-0.09 ; 1.5) | 0.026 | 0.78 (0.11; 1.46) | 0.022 |
| Arm 1 | | 0.26 (-0.42 ; 0.94) | 0.45 | 0.26 (-0.41 ; 0.93) | 0.45 |
| Time | | -0.014 (-0.022; -0.006) | 0.001 | -0.014 ( -0.022; -0.006) | 0.001 |
| Arm 3 * Time | | ref | - | ref | - |
| Arm 2 * Time | | -0.007 (-0.005 ; 0.018) | 0.26 | 0.07 (-0.005 ; 0.018) | 0.26 |
| Arm 1 * Time | | 0.014 (-0.002; 0.027) | 0.024 | 0.014 (0.002 ; 0.027) | 0.024 |

**Bone Mineral Density**

| **Patients** | **59** |  |  |  |  |
| --- | --- | --- | --- | --- | --- |
| **X rays** | **116** |  |  |  |  |
| **C Bone Mineral Density adjusted for symptom duration** | | | | | |
|  | | **Adjusted** |  | **Unadjusted** |  |
|  | | **Β (95% CI)** | **P-value** | **Β (95% CI)** | **P-value** |
| Arm 3 | | ref | - | ref | - |
| Arm 2 | | 0.24 (-0.45 ; 0.94) | 0.49 | 0.28 (-0.41 ; 0.98) | 0.42 |
| Arm 1 | | 0.74 (0.077 ; 1.41) | 0.030 | 0.74 (0.07 ; 1.41) | 0.031 |
| Time | | 0.027 (-0.017 ; 0.038) | <0.001 | 0.027 | <0.001 |
| Arm 3 * Time | | Ref | - | ref | - |
| Arm 2 * Time | | -0.009 (-0.023 ; 0.006) | 0.26 | -0.009 (-0.02 ; 0.006) | 0.25 |
| Arm 1 * Time | | -0.028 (-0.043 ; -0.013) | <0.001 | -0.028 (-0.043 ; -0.012) | <0.001 |

LMM: linear mixed model, arm 1: initial sequential monotherapy, arm 2 initial MTX with prednisolone bridging 6 weeks, arm 3 initial MTX with etanercept. BMD Bone Mineral Density; B= β ; 95%CI: 95% Confidence Interval.
