# Supplementary material for: No radiographic wrist damage after treatment to target in recent-onset juvenile idiopathic arthritis
Source: Pediatr Rheumatol Online J. 2019 Sep 4;17:62. doi: 10.1186/s12969-019-0362-1 (PMC6727344; doi:10.1186/s12969-019-0362-1)
Supplement: Supplementary file 4 — A Sensitivity analysis of patients with wrist arthritis, without n = 6 with never wrist arthritis. B Sensitivity analysis of patients with polyarticular JIA. C LMM for mean JADAS10 score in relation to Poznanski, BA and BMD. (DOCX 804 kb) [file 12969_2019_362_MOESM4_ESM.docx]

**Additional file 4**

**A Sensitivity Analysis patients with wrist arthritis, without n=6 with never wrist arthritis**

**Table, LMM for Poznanski, BA and BMD**

**Poznanski adjusted for age and symptom duration**

| Patients | 54 |  |  |
| --- | --- | --- | --- |
| X rays | 105 |  |  |
|  | | **Β (95% CI)** | **P** |
| Arm 3 | | ref | - |
| Arm 2 | | 0.076 (-0.43; 0.58) | 0.771 |
| Arm 1 | | 0.16 (-0.30; 0.61) | 0.498 |
| Time | | 0.0036 (-0.0047; 0.012) | 0.391 |
| Arm 3 * Time | | ref | - |
| Arm 2 * Time | | -0.0058 (-0.018; 0.0066) | 0.359 |
| Arm 1 * Time | | -0.0058 (-0.018; 0.0061) | 0.337 |

**Bone Age adjusted for symptom duration**

|  | | | | |
| --- | --- | --- | --- | --- |
| Patients | 37 |  |  |  |
| X rays | 72 |  |  |  |
|  | | **Β (95% CI)** | **P** |  |
| Arm 3 | | ref | - |  |
| Arm 2 | | 0.99 (0.31; 1.68) | 0.005 |  |
| Arm 1 | | 0.34 (-0.31; 0.98) | 0.304 |  |
| Time | | -0.014 (-0.022; -0.0053) | 0.001 |  |
| Arm 3 * Time | | Ref |  |  |
| Arm 2 * Time | | 0.0092 (-0.0029; 0.021) | 0.136 |  |
| Arm 1 * Time | | 0.014 (0.0016; 0.027) | 0.027 |  |

**Bone Mineral Density adjusted for symptom duration**

| Patients | 53 |  |  |
| --- | --- | --- | --- |
| X rays | 104 |  |  |
|  | | **Β (95% CI)** | **P** |
| Arm 3 | | ref | - |
| Arm 2 | | 0.077 (-0.67; 0.82) | 0.841 |
| Arm 1 | | 0.84 (0.16; 1.51) | 0.015 |
| Time | | 0.028 (0.017; 0.039) | <0.001 |
| Arm 3 * Time | | ref | - |
| Arm 2 * Time | | -0.0054 (-0.022; 0.011) | 0.524 |
| Arm 1 * Time | | -0.029 (-0.045; -0.013) | <0.001 |

LMM linear mixed model, arm 1: initial sequential monotherapy, arm 2 initial MTX with prednisolone bridging 6 weeks, arm 3 initial MTX with etanercept. BMD Bone Mineral Density; B: β ; 95%CI: 95%Confidence Interval

**Additional file 4 B Sensitivity Analysis Polyarticular JIA patients only**

**Table, LMM for Poznanski, BA and BMD adjusted for age and/or symptom duration**

**Poznanski**

| **Patients** | 50 |  |  |
| --- | --- | --- | --- |
| **X rays** | 99 |  |  |
|  | | **Β (95% CI)** | **P-value** |
| Arm 3 | | ref | - |
| Arm 2 | | 0.227 (-0.035-0.81) | 0.444 |
| Arm 1 | | 0.219 (-0.34-0.77) | 0.44 |
| Time | | 0.0035 (-0.0049; 0.012) | 0.415 |
| Arm 3 * Time | | ref | - |
| Arm 2 * Time | | -0.0041 (-0.016 ; 0.0075) | 0.493 |
| Arm 1 * Time | | -0.0042 (-0.016; 0.0078) | 0.491 |

**Bone age adjusted for symptom duration**

| **Patients** | 34 |  |  |
| --- | --- | --- | --- |
| **X rays** | 68 |  |  |
|  | | **Β (95% CI)** | **P-value** |
| Arm 3 | | Ref | - |
| Arm 2 | | 0.89 (0.18;1,61) | 0.015 |
| Arm 1 | | 0.45 (-0.28 ; 1.17) | 0.225 |
| Time | | -0.012 (-0.023; -0.0017) | 0.023 |
| Arm 3 * Time | | Ref | - |
| Arm 2 * Time | | 0.0086 (-0.0056; 0.023) | 0.24 |
| Arm 1 * Time | | 0.015 (-0.0003; 0.03) | 0.055 |

**Bone Mineral Density adjusted for symptom duration**

| **Patients** | 50 |  |  |
| --- | --- | --- | --- |
| **X rays** | 100 |  |  |
|  | | **Β (95% CI)** | **P-value** |
| Arm 3 | | ref | - |
| Arm 2 | | 0.37 (-0.38 ; 1.12) | 0.33 |
| Arm 1 | | 0.64 (-0.08 ; 1.36) | 0.082 |
| Time | | 0.027 (0.015 ; 0.038) | 0 |
| Arm 3 * Time | | Ref | - |
| Arm 2 * Time | | -0.10 (-0.026 ; 0.0053) | 0.20 |
| Arm 1 * Time | | -0.026 (-0.043 ; -0.010) | 0.001 |

LMM linear mixed model, arm 1: initial sequential monotherapy, arm 2 initial MTX with prednisolone bridging 6 weeks, arm 3 initial MTX with etanercept. BMD Bone Mineral Density; B: β ; 95%CI: 95%Confidence Interval

**Additional file 4C MeanJADAS10score over time**

**Poznanski adjusted for age and symptom duration**

| Patients | 60 |  |  |
| --- | --- | --- | --- |
| X rays | 117 |  |  |
|  | | **Β (95% CI)** | **P-value** |
| meanJADAS10 * Time | | 0.0010 (-0.00038-0.0024) | 0.15 |

**Bone Age adjusted for symptom duration**

| Patients | 41 |  |  |
| --- | --- | --- | --- |
| X rays | 80 |  |  |
|  | | **Β (95% CI)** | **P-value** |
| meanJADAS10 * Time | | -0.00017 (-0.0018-0.0014) | 0.84 |

**Bone Mineral Density adjusted for symptom duration**

| Patients | 59 |  |  |
| --- | --- | --- | --- |
| X rays | 116 |  |  |
|  | | **Β (95% CI)** | **P-value** |
| meanJADAS10 * Time | | 0.00069 (-0.0012-0.0026) | 0.48 |

LMM linear mixed model; meanJADAS10: mean Juvenile Arthritis Disease Activity Score with up to max 10 joints, over 2 years’ time; B: β ; 95%CI: 95%Confidence Interval

Predicted Z-scores RM/M2 (Poznanski score) over time, for different meanJADAS10 scores


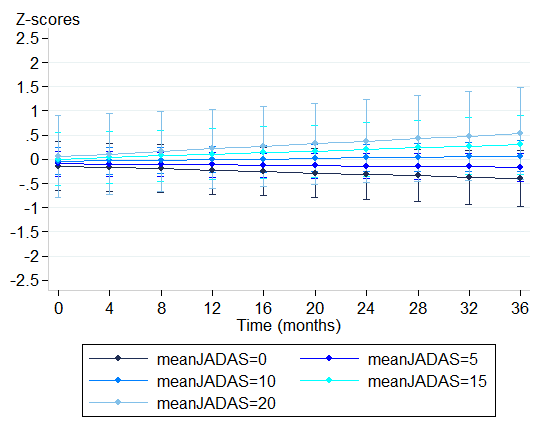


Predicted Z-scores for Bone Age over time for different meanJADAS10scores


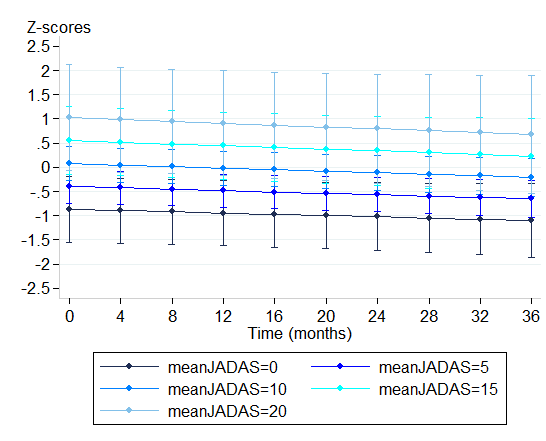


Predicted Z-scores for Bone Mineral Density over time for different meanJADAS10scores
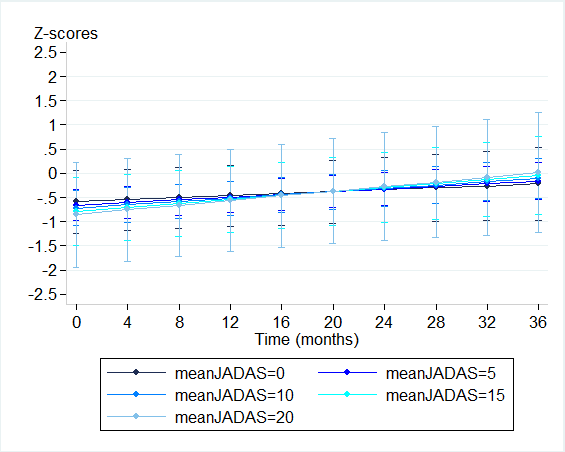


All predictions are from Linear Mixed Models, corrected for age and symptom duration for Poznanski score, corrected for symptom duration for Bone Age and Bone Mineral Density
